# Avoiding pitfalls in diagnosing basilar artery occlusive disease: clinical and imaging clues — case report

**DOI:** 10.1590/S1516-31802010000300009

**Published:** 2010-05-06

**Authors:** Adriana Bastos Conforto, Jovana Gobbi Marchesi Ciríaco, Fábio Iuji Yamamoto, Paulo Puglia, Claudia da Costa Leite, Maria da Graça Morais Martin, Milberto Scaff

**Affiliations:** I MD, PhD. Neurologist, Neurology Division, Hospital das Clínicas (HC), Universidade de São Paulo (USP), and researcher at Instituto Israelita de Ensino e Pesquisa Albert Einstein (IIEPAE), São Paulo, Brazil.; II Postgraduate student, Department of Neurology, Universidade de São Paulo (USP), São Paulo, Brazil.; III MD. Neurologist, Neurology Division, Hospital das Clínicas (HC), Universidade de São Paulo (USP), São Paulo, Brazil.; IV MD, PhD. Radiologist, Department of Radiology, Hospital das Clínicas (HC), Universidade de São Paulo (USP), São Paulo, Brazil.; V MD, PhD. Full professor, Department of Radiology, Hospital das Clínicas (HC), Universidade de São Paulo (USP), São Paulo, Brazil.; VI MD, PhD. Head, Department of Neurology, Hospital das Clínicas (HC), Universidade de São Paulo (USP), São Paulo, Brazil.

**Keywords:** Stroke, Carotid artery diseases, Basilar artery, Hemiparesis, Brain stem infarctions, Acidente cerebral vascular, Doenças das artérias carótidas, Artéria basilar, Hemiparesia, Infartos do tronco encefálico

## Abstract

**CONTEXT:**

The aim of this paper was to report on the characteristics that aid in establishing the diagnosis of basilar artery occlusive disease (BAOD) among patients with hemiparesis and few or minor symptoms of vertebrobasilar disease.

**CASE REPORT:**

This report describes two cases in a public university hospital in São Paulo, Brazil. We present clinical and imaging findings from two patients with hemiparesis and severe BAOD, but without clinically relevant carotid artery disease (CAD). One patient presented transient ischemic attacks consisting of spells of right hemiparesis that became progressively more frequent, up to twice a week. The neurological examination revealed slight right hemiparesis and right homonymous hemianopsia. Magnetic resonance imaging (MRI) revealed pontine and occipital infarcts. Magnetic resonance angiography and digital subtraction angiography revealed severe basilar artery stenosis. The other patient presented sudden left-side hemiparesis and hypoesthesia. One year earlier, she had reported sudden onset of vertigo that, at that time, was attributed to peripheral vestibulopathy and was not further investigated. MRI showed a right-side pontine infarct and an old infarct in the right cerebellar hemisphere. Basilar artery occlusion was diagnosed. Both patients presented their symptoms while receiving aspirin, and became asymptomatic after treatment with warfarin.

**CONCLUSIONS:**

Misdiagnosing asymptomatic CAD as the cause of symptoms in BAOD can have disastrous consequences, such as unnecessary carotid endarterectomy and exposure to this surgical risk while failing to offer the best available treatment for BAOD. Clinical and imaging features provided important clues for diagnosis in the cases presented.

## RESUMO

**CONTEXTO:**

O objetivo deste artigo foi de relatar as características que auxiliam no estabelecimento do diagnóstico da doença oclusiva da artéria basilar (DOAB) em pacientes com hemiparesia e poucos sintomas, ou sintomas leves de doença vertebrobasilar.

**RELATO DE CASO:**

Descrição de dois casos em um hospital público universitário em São Paulo, Brasil. Apresentamos características clínicas e radiológicas de dois pacientes com hemiparesia e DOAB grave, na ausência de doença carotídea (DCA) relevante. Um paciente apresentou ataques isquêmicos transitórios consistindo de hemiparesia direita transitória, que se tornaram progressivamente mais frequentes, até duas vezes por semana. O exame neurológico revelou hemiparesia direita e hemianopsia homônima direita. A ressonância magnética (RM) revelou infartos pontinos e occipitais. As angiografias por ressonância magnética e por subtração digital revelaram estenose grave da artéria basilar. Outra paciente apresentou hemiparesia e hipoestesia de instalação súbita, à esquerda. Um ano antes, ela havia apresentado vertigem súbita, na ocasião atribuída a vestibulopatia periférica, não investigada em maior profundidade. A RM mostrou infarto pontino à direita e infarto antigo no hemisfério cerebelar direito. Foi diagnosticada oclusão da artéria basilar. Ambos os pacientes apresentaram os sintomas enquanto faziam tratamento com aspirina, e se tornaram assintomáticos após tratamento com warfarina.

**CONCLUSÕES:**

O diagnóstico errôneo de DCA como causa de sintomas na DOAB pode ter conseqüências desastrosas, como a endarterectomia carotídea desnecessária e a exposição a tal risco cirúrgico, ao invés de oferecimento do melhor tratamento disponível para a DOAB. Características clínicas e radiológicas forneceram pistas importantes para o diagnóstico nos casos apresentados.

## INTRODUCTION

Traditionally, large-artery vertebrobasilar disease has been associated with symptoms resulting from brainstem, cerebellar, thalamic or occipital involvement. However, basilar artery occlusive disease (BAOD) may present predominantly with hemiparesis, and even mimic the lacunar syndrome of pure motor hemiparesis that is frequently associated with lesions in the internal carotid artery (ICA) region.^1-3^ It is important to establish the correct etiology of motor deficits before performing carotid endarterectomy in patients with hemiparesis, because the treatment for BAOD differs from that of carotid artery disease (CAD).

We describe two patients who complained of hemiparesis and had severe BAOD but without clinically relevant CAD.

## CASE REPORTS

**Figure 1.**
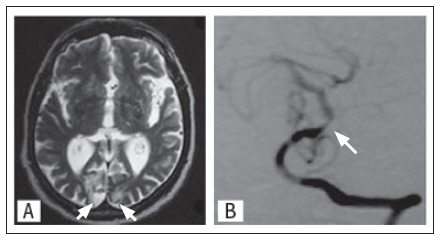
A. T2-weighted magnetic resonance imaging from Case 1, showing bilateral occipital infarcts (arrows). B. Digital subtraction angiography showing severe basilar artery stenosis (arrow).

**Patient 1:** A 73-year-old man presented with transient ischemic attacks (TIAs) consisting of stereotyped spells of right hemiparesis lasting from five to ten minutes, which became progressively more frequent, up to twice a week. Eight years earlier, he had reported sudden onset of right hemiparesis and hemihypoesthesia that improved over a few days. Two years before the present report, he had presented sudden onset of vertigo and "bilateral visual blurring"; computed tomography (CT) showed a right occipital infarct that, at that time, was not investigated. He had been on aspirin for the past six years and had a history of arterial hypertension, diabetes mellitus, ischemic heart disease, peripheral artery disease and dyslipidemia. The neurological examination revealed slight right hemiparesis and right homonymous hemianopsia. Cervical Doppler showed 60-70% stenosis of the left carotid bulb. Magnetic resonance imaging (MRI) showed old infarcts in the right pons and bilateral occipital lobes (Figure 1). The right pontine and the right occipital infarcts were asymptomatic. There were no lesions in the ICA region. Magnetic resonance angiography (MRA) showed basilar artery stenosis but no critical stenosis in the left ICA. Digital subtraction angiography (DSA) demonstrated severe (80%) mid-basilar artery stenosis (Figure 1), 60% stenosis at the origin of the left vertebral artery origin, 70% stenosis at the origin of the right vertebral artery origin and 30% stenosis in both cervical ICAs. Warfarin was prescribed and the patient did not have any further symptoms.

**Figure 2.**
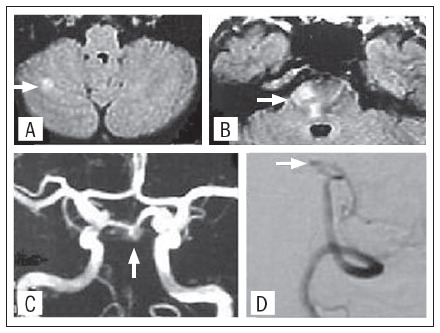
Fluid attenuated inversion recovery images showing acute infarcts (arrows) in the right cerebellar hemisphere (A) and right pons (B) of Case 2. Magnetic resonance angiography showing absence of basilar artery flow signal (C) suggestive of occlusion (arrow), which was confirmed by digital subtraction angiography (arrow, D).

**Patient 2:** A 55-year-old woman presented sudden onset of left hemiparesis lasting for more than 24 hours. One year earlier, she had reported vertigo that, at that time, was attributed to peripheral vestibulopathy. The neurological examination showed left hemiparesis and left hypoesthesia. Fluid-attenuation inversion-recovery and diffusion-weighted images revealed an acute right pontine infarct and an old infarct in the right cerebellar hemisphere (Figure 2). MRA suggested and DSA confirmed occlusion of the middle portion of the basilar artery. There were no abnormalities in other cervicocerebral arteries and a transthoracic echocardiogram was normal. The patient continued to have TIAs during the first month after the ictus, in spite of using aspirin 300 mg/day, but became asymptomatic after aspirin was switched to warfarin.

Both patients participated in this study on the natural history of BAOD. The protocol was approved by the local Ethics Committee and the patients provided informed consent for their participation.

## DISCUSSION

These cases illustrate the association between hemiparesis and BAOD. In Case 1, Doppler findings raised the possibility of performing endarterectomy to treat an ICA stenosis presenting with transient right hemiparesis. However, DSA showed that the left ICA only presented 30% stenosis, while the basilar artery was severely stenosed. MRI revealed previous infarcts in the vertebrobasilar region, but not in the ICA region. After anticoagulation, the symptoms disappeared. In Case 2, replacement of antiplatelet therapy with anticoagulation also led to symptom resolution.

The frequency of pure hemiparesis is 7 to 9% in stroke records.^1,2^ Pure hemiparesis may occur when a pontine lesion forms in the path of the corticospinal tracts but not of the cranial nerves or cerebellar or sensory pathways. Cases of capsular lacunae and large hemispheric lesions in the ICA region may also present with sensory-motor stroke,^4^ and the clinical findings may not reliably distinguish between them.^2^

Even though pontine ischemic lesions presenting with hemiparesis may clinically mimic a lacunar syndrome, they may be caused by different pathogenetic mechanisms,^4^ such as small artery disease (SAD), basilar artery branch disease (BABD) or BAOD.^3^ SAD is usually attributed to lesions of small penetrating arteries caused by lipohyalinosis and fibrinoid degeneration. The ostia of branching arteries can become stenosed or occluded due to microatheromas or large atheroma plaque in BAOD cases. Hemodynamic insufficiency and embolism caused by BAOD or other sources may also cause pontine infarcts.^2,3^

Pontine infarcts following endarterectomy have been reported in patients with TIAs characterized by hemiparesis and normal CT scans,^2^ thus indicating that vertebrobasilar disease may have been the untreated cause of hemiparesis in these patients. In the cases presented here, the TIAs stopped after anticoagulation. Even though there are no clear evidence-based guidelines favoring treatment with warfarin for patients with BAOD, anticoagulants may be potentially more effective than antiplatelet agents under these conditions.^4,5^ Endovascular treatment with angioplasty and stenting is considered to be an investigational technique, but may be considered on an individual basis for patients who have symptoms despite medical therapy.^6-9^

BAOD may lead to severe neurological disability, in association with tetraparesis, decreased level of consciousness, "locked-in" syndrome or other classical vertebrobasilar neurological syndromes. However, milder clinical presentations also occur and, in some patients, they precede neurological deficits of greater severity.^10^ Neuroimaging is mandatory in suspected cases. Transcranial Doppler ultrasound and magnetic resonance angiography noninvasively identify 50 to 99% of intracranial large vessel stenoses with substantial negative predictive value (85% for transcranial Doppler and 87% for magnetic resonance angiography). These tests can therefore reliably exclude the presence of intracranial stenosis. Abnormal results from these tests require a confirmatory test such as angiography.^11^

In order to provide the best available treatment for patients with hemiparesis and BAOD, as well as to avoid misdiagnosis of CAD and unnecessary surgical procedures, the correct diagnosis has to be made. Pontine infarcts may not be diagnosed on CT scans, even in patients presenting with hemiparesis lasting longer than one day. In addition, previous TIAs or infarcts presenting with vertigo, ataxia or other symptoms are often present in patients with hemiparesis caused by vertebrobasilar disease,^2^ as in our patients. These symptoms may be subtle and go unnoticed. Such symptoms should be considered red flags and be actively investigated in patients presenting with hemiparesis.

Neuroimaging is another important tool for guiding clinical judgment. MRI performed in case 1 showed multiple ischemic lesions affecting the vertebrobasilar region, but not the ICA region. Thus, the neurological examination and MRI may indicate that the complaint of pure hemiparesis may actually not represent true pure hemiparesis, and that other clinical features or lesions may be present. These important clues should not be ignored when making therapeutic decisions regarding clinical or surgical treatment.
